# From taxonomy to metabolic output: what factors define gut microbiome health?

**DOI:** 10.1080/19490976.2021.1907270

**Published:** 2021-04-23

**Authors:** Tomasz Wilmanski, Noa Rappaport, Christian Diener, Sean M. Gibbons, Nathan D. Price

**Affiliations:** a Institute for Systems Biology, Seattle, WA, USA; b eScience Institute, University of Washington, Seattle, WA; c Department of Bioengineering, University of Washington, Seattle, WA, USA; d Onegevity Health, New York, NY, USA

**Keywords:** Gut microbiome, metabolomics, therapeutics, precision medicine

## Abstract

Many studies link the composition of the human gut microbiome to aberrant health states. However, our understanding of what constitutes a ‘healthy’ gut ecosystem, and how to effectively monitor and maintain it, are only now emerging. Here, we review current approaches to defining and monitoring gut microbiome health, and outline directions for developing targeted ecological therapeutics. We emphasize the importance of identifying which ecological features of the gut microbiome are most resonant with host molecular phenotypes, and highlight certain gut microbial metabolites as potential biomarkers of gut microbiome health. We further discuss how multi-omic measurements of host phenotypes, dietary information, and gut microbiome profiles can be integrated into increasingly sophisticated host-microbiome mechanistic models that can be leveraged to design personalized interventions. Overall, we summarize current progress on defining microbiome health and highlight a number of paths forward for engineering the ecology of the gut to promote wellness.

## Introduction

The human gut is inhabited by a diverse set of bacteria, archaea, viruses, protists and fungi, which are collectively referred to as the human gut microbiome. The large number of genes that comprise the gut metagenome surpasses that of the human genome by at least three orders of magnitude.^1^ Consequently, the gut microbiome contains a diverse array of biological and metabolic functions, many of which modulate host phenotypes and fitness.^2^ Most of the microbial biomass in the gut is bacterial, which is the primary focus of this review. Over the last decade, gut bacterial composition has been linked to a number of human diseases, extending the impact of the gut microbiome on host health beyond just gastrointestinal disorders. Gut bacteria and their metabolic products have been shown to modulate host immunity,^3^ regulate metabolic homeostasis,^4^ and influence neurocognitive function.^5^ Despite an emergent understanding of gut microbial influence in a number of host disease pathologies, we are only now beginning to unravel what constitutes a healthy microbiome-host relationship. Importantly, identifying a healthy gut microbiome, and how to effectively monitor it, are essential steps toward designing microbiome-based interventions for treating disease and promoting human wellness.

Several challenges remain in defining a healthy gut microbiome. Variability across geography is a major obstacle in translating gut microbiome findings across populations, with dominant species in the gut showing a large degree of heterogeneity across cultures and continents.^6–8^ Indeed, significant microbial heterogeneity is even present in individuals living just a few hundred miles apart, leading some to call for geo-localized microbiome disease models.^9^ This taxonomic variability indicates a high degree of functional redundancy across human gut microbes, giving rise to myriad taxonomic compositions that result in comparable ecosystem function.

Unknown impacts of drugs and diet on the gut microbiome represent additional obstacles to defining and monitoring microbiome health. Not only can a wide variety of drugs be metabolized by the gut microbiota into potentially bioactive compounds,^10^ drugs can also directly modulate gut microbial composition and function.^11,12^ Dietary patterns strongly influence and shape the gut microbiome,^13,14^ while gut microbial composition may also modulate host metabolic responses to specific dietary components.^15^ These interactions are further complicated by significant interindividual variability in gut microbial composition at the level of relative abundance of individual taxa,^16^ presence of specific bacterial strains, and variability in structural variants within individual genes.^17,18^ Collectively, all these factors underscore the need for personalized assessments of gut community function, host diet/lifestyle/behavior, and host molecular phenotypes when attempting to define a healthy gut ecosystem.

Recent advancements in microbiome research have enabled the identification of more translatable markers of gut microbiome function, and provide paths toward rationally engineering the functional output of the gut ecosystem to promote human health. In the following sections, we provide an overview of current approaches to defining microbiome health, focusing primarily on adults. We further characterize potential biomarkers that can be used to monitor the gut ecosystem, and discuss future directions for how the gut microbiome can be manipulated to promote human wellness. In particular, while monitoring gut microbial taxonomic and metagenomic profiles can serve as a useful marker for certain microbiome perturbations, we highlight alternative methods for quantifying microbiome health through a host lens, particularly by focusing on small molecular weight microbial products, which have the potential to result in more reproducible signals that translate readily across vastly diverse cohorts and disease states.^19^ Finally, we discuss how the use of sophisticated mechanistic models that integrate gut metagenomics with host physiological and lifestyle data may provide key insights into how we might engineer individual microbiomes to promote human health.

## Defining microbiome health

While a strict definition of a healthy gut microbiome remains elusive, several key features have been proposed. These include ecological principles of system stability that are important to a healthy gut ecosystem. For example, ecological resilience refers to the ability of an ecosystem to return to its original state following a perturbation, while ecological resistance refers to the ability of an ecosystem to resist a perturbation in the first place.^20–22^ Thus, a healthy gut microbiome could be defined as one that can defend itself against e.g. invasion by opportunistic pathogens (resistance), and one that can readily recover to its original state after e.g. routine antibiotic treatment (resilience). Because there are many combinations of gut commensal taxa that result in a healthy, stable ecosystem, longitudinal monitoring of individuals is likely important for tracking deleterious changes in resistance and resilience. However, host phenotypic context is also essential when defining healthy stability. For example, recurrent *Clostridioides difficile* (rCDI) infections can be highly resilient to antibiotic treatment, but this resilience works counter to host health.^23^ Similarly, gut microbiome composition is highly dynamic in the first three years of human life.^24^ This high level of instability is likely a combination of changing dietary inputs (i.e. termination of breast feeding, transition into solid foods, etc.) and immune system maturation,^25^ and thus cannot be interpreted within the same health context as changes in an adult microbiome.

Microbiome health is largely determined by the diverse pool of compounds produced by the gut microbiota that regulate ecosystem dynamics and influence host physiology. In addition to providing specific nutrients to the host, microbial metabolic output has increasingly been implicated in a diverse array of biological functions, both locally in the gastrointestinal tract and distally across a variety of host organs through absorption in the blood.^26,27^ This functional component of microbiome health can be inferred through modeling the metabolic capacity of the gut metagenome, transcriptome, and proteome, or where possible, determined by directly measuring the microbial metabolic products themselves in blood and stool.

Thus, a healthy gut microbial composition can be thought of as one that successfully maintains long-term stability, resists invasive pathogens, supplies key nutrients (including vitamins and fermentation byproducts) to its host, and helps maintain host metabolic and immunological homeostasis. A disruption to any of these functions is consequently indicative of an unhealthy gut microbial state. In the following sections, we describe specific approaches to monitoring these key aspects of gut microbiome function.

## Taxonomic signatures of gut microbiome health

Early efforts toward characterizing the human gut microbiome hypothesized there may be a set of ‘core’ taxa that are ubiquitously present across healthy adult individuals.^28,29^ With a ‘core’ phylogenetic profile as a reference point, any deviation from this composition could be considered an unhealthy perturbation. However, given the vast diversity in gut microbiome composition reported between healthy individuals over the last decade, there is little evidence for such a strict taxonomic core.^8,16^ Indeed, there appear to be myriad taxonomic compositions that support a healthy, functional microbiome-host relationship across diverse populations.

Additional insight into taxonomic markers of microbiome health comes from investigating consistent microbial differences associated with diseases. At the taxonomic level, gut microbial ‘dysbiosis’ appears to fall into several qualitatively different categories: 1) enrichment of a small number of putatively detrimental taxa, 2) depletion of a small number of putatively beneficial taxa, and 3) a complete ecosystem turnover.^30^

A common example of ‘dysbiosis’ of the first type is associated with colorectal cancer (CRC), where taxa normally found only in the oral cavity (e.g. *Fusobacterium* or *Porphyromonas)* are consistently enriched in CRC patients relative to healthy controls.^31–33^ *Fusobacterium nucleatum*, in particular, was shown to directly promote tumor growth and disease progression in animal models of CRC through recruitment of tumor-infiltrating immune cells and promoting a pro-inflammatory tumor microenvironment.^34,35^ *F. nucleatum* also directly contributes to cancer cell immune evasion by interacting with natural killer (NK) cells, inhibiting their activity via binding of the Fap2 protein to the NK cell inhibitory receptor TIGIT.^36^ Thus, although relatively few causal links between specific gut taxa and disease have been identified to date, in certain rare cases, screening for particular microbes may be a useful strategy for effectively monitoring gut health in high-risk populations.

Chronic metabolic and immune-related disease states often fall into the second category of ‘dysbiosis’, where a small number of beneficial microbes are depleted from the ecosystem. For example, inflammatory bowel disease (IBD), including both ulcerative colitis (UC) and Crohn’s disease (CD), shows a consistent depletion of putatively beneficial short-chain-fatty-acid (SCFA) producing taxa (e.g. *Faecalibacterium, Roseburia, Ruminococcus*).^37–39^ Similar declines in SCFA producers, particularly *Faecalibacterium*, have been reported in a variety of human disorders, including nonalcoholic fatty liver disease and bipolar disorder.^40,41^ The beneficial role of *Faecalibacterium* in the gut is in part attributed to its anti-inflammatory potential. Studies in mice have demonstrated that administration of *Faecalibacterium prausnitzii* can reduce chemically-induced colitis, decrease production of inflammatory cytokines including IL-6 and IFNγ, and restore proper gut barrier function.^42^ Because of its consistent depletion across disease states, *F. prausnitzii*, in particular, has been proposed as a biomarker for monitoring gut microbiome health.^43^

Similar to *F. prausnitzii*, the mucin degrading commensal *Akkermansia muciniphila* has emerged as a key component of a healthy gut ecosystem, with its depletion observed in UC^44,45^ and under conditions of compromised metabolic health (*e.g*. insulin resistance, hyperglycemia).^46,47^ The beneficial effects of *A. muciniphila* stem in part from its ability to support gut barrier integrity and decrease inflammation.^48^ Administration of *A. muciniphila* in mice increased intestinal levels of endocannabinoid signaling glycerolipids 2-oleoylglycerol and 2-arachidonoylglycerol, which stimulate gut peptide release, which in turn serves to improve host energy homeostasis and gut barrier function.^49^ Recent studies have further demonstrated that supplementation with *A. muciniphila* is sufficient to improve several markers of metabolic health in animal models and humans, including total cholesterol, fasting blood glucose, and markers of insulin sensitivity and liver function.^50,51^ Taken together, these findings indicate that a healthy gut microbial composition consists of a minimal set of anaerobic fiber-fermenting and mucin-degrading taxa at relatively high levels of abundance. Consequently, depletion of these taxa from the ecosystem reflects, and potentially contributes to, disease-associated states.

The third type of ‘dysbiosis’ -a complete ecosystem turnover – is often the result of a severe environmental perturbation. These perturbations include antibiotics treatment or enteric infections. In these cases, there is often both a rise in the relative abundance of facultative anaerobes and a decrease in the relative abundance of strictly anaerobic, fiber-degrading commensals.^21^ Such large-scale turnover in the gut ecosystem is often associated with severe gastrointestinal symptoms, like abdominal pain and diarrhea, which can be assessed without the need for microbiome sequencing.

## Diversity metrics as markers of microbiome health

Abstract metrics that summarize multivariate gut microbiome profiles into a single value can overcome some of the obstacles intrinsic to defining microbiome health based on abundances of individual taxa, and provide further insight into the overall structure and stability of the gut ecosystem.

α*-diversity metrics*: α-diversity is a measure of within-sample ecological diversity and is one of the most commonly reported results in gut microbiome research. A number of different metrics exist for summarizing α-diversity, including the number of taxa present in the ecosystem (e.g. richness), the evenness of the taxon abundance distributions (e.g. Pielou’s evenness index),^52^ measures that incorporate phylogenetic diversity (e.g. PD Wholetree),^53^ and measures summarizing both taxon richness and evenness (e.g. Shannon entropy).^54^ Depletion of gut α-diversity has been associated with a number of diseases including IBD^55^ and type II diabetes,^56^ but most consistently with enteric infections^57^ and antibiotics use.^58^ Low α-diversity can often be a more consistent marker of a major gut microbial perturbation than changes in the abundances of specific taxa, particularly across geographically distinct populations. For example, UC and CD have both been characterized by decreased gut α-diversity across nationally distinct cohorts, while taxonomic differences showed less consistency across the same populations.^59^

The impact of disease and environmental factors on gut α-diversity is often transient. Even a perturbation as large as antibiotic treatment has been shown to only temporarily deplete gut α-diversity.^60,61^ Following the cessation of antibiotic treatment, α-diversity generally returns to its original level within weeks-to-months. This makes α-diversity a particularly attractive metric for monitoring gut microbiome health, since it can capture both long-term structure and short-term responses to perturbations. Although not specific to any one disease, a depletion in gut α-diversity may serve as a warning sign that the gut ecosystem is compromised.

Higher α-diversity is often thought to reflect a healthier gut ecosystem. This is supported by depletion of α-diversity in several disease states, as discussed above, as well as a higher risk of rCDI with lower baseline α-diversity.^62^ This increased risk of infection is likely due to the availability of metabolic niches normally saturated by commensals in more diverse microbiomes. Opportunistic pathogens, such as *C. difficile* or enteropathogenic *Escherichia coli*, can exploit these niches.^63^ However, there may be an upper threshold to gut α-diversity, beyond which a more diverse microbiome could be detrimental to human health. For example, constipation has been associated with a more diverse microbiome across several studies.^64,65^ Our research group and others have further shown that measures of gut α-diversity correlate positively with both constipation and a number of microbial protein fermentation products in the blood, such as *p*-cresol sulfate and phenylacetylglutamine.^66,67^ At high enough concentrations, these compounds may be toxic to the host, resulting in damage to host organs including the kidneys and heart.^68,69^

There likely exists an optimal range for gut α-diversity, although this range may vary depending on the set of dominant taxa that populate the gut (e.g. *Prevotella* vs. *Bacteroides* dominated individuals).^70^ This optimal range may be further dependent on the specific health and physiological state of the human host. For example, a number of microbial protein fermentation products positively associated with gut α-diversity have been linked to worse outcomes in chronic kidney disease (CKD) patients (*p*-cresol sulfate, Trimethylamine N-oxide (TMAO), and phenylacetylglutamine).^66,71^ These microbial metabolites can act as cardiac and uremic toxins, particularly in a host whose ability to efficiently filter and excrete these compounds is compromised. Thus, lowering gut α-diversity in CKD patients through, for example, decreasing stool transit time (i.e. increasing fiber intake or taking laxatives) could be a potential therapeutic strategy aimed at slowing the progression of the disease. Such interventions may be particularly successful, as constipation is one of the most commonly reported gastrointestinal disorders among CKD patients.^72^ Similar personalized ranges of gut α-diversity may be especially important in older populations, where the risk of enteric infections is particularly high and organ function is in decline.^73^ In this case, an optimal gut α-diversity would likely be one high enough to protect against gastrointestinal infections, while also being low enough to minimize the burden of mildly toxic protein fermentation byproducts on an aging host.

*ß-diversity metrics*: ß-diversity captures inter-individual variability in the composition of the gut microbiome (i.e. how dissimilar is one sample from another), and is commonly reported alongside α-diversity in microbiome studies. Similar to α-diversity, ß-diversity integrates a large amount of information about community composition into a single, abstract metric. Several ß-diversity measures exist, including unweighted measures such as Jaccard and unweighted UniFrac,^74^ which capture differences in the presence and absence of different taxa between two samples, and weighted measures such as Bray-Curtis^75^ and weighted UniFrac,^76^ which capture compositional differences between samples weighted by taxon abundances.

The application of ß-diversity analyses to monitoring gut microbiome health shows the greatest promise when applied longitudinally. When measured across time, each individual serves as their own control (comparing their recent sample to previous samples), which allows researchers to account for high interindividual variability in gut microbiome composition, while also capturing the rate of drift from an initial starting point. Previous studies of healthy adults have shown a relatively high level of temporal stability in gut microbiome composition, with individuals harboring the same taxa for years or even decades.^77–79^ Perhaps unsurprisingly, temporal stability and gut microbial α-diversity tend to be related.^80^ Shannon diversity was positively associated with gut microbiome stability (assessed using Weighted UniFrac) in healthy adults when the microbiome was sampled weekly across the course of three months.^81^ Similarly, very low gut microbial α-diversity in a cohort of older individuals corresponded to higher levels of ß-diversity instability when measured up to six months apart.^82^ Furthermore, like with low α-diversity, a high level of ß-diversity instability can be indicative of disease states. For example, both fatty liver disease and type II diabetes were associated with higher gut ß-diversity instability (Bray-Curtis dissimilarity) in a cohort of German adults when sampled across a span of five years.^80^ Using 16S rRNA gene sequencing, Halfvarson *et al*.^83^ demonstrated that patients suffering from IBD (UC and CD) had higher levels of temporal instability compared to healthy controls. Interestingly, some IBD patients in that study appeared to transition readily between health and disease-associated states. Thus, if sampled at a single time point, gut microbial perturbations in an IBD patient could go unnoticed. The major advantage of longitudinal ß-diversity analysis in defining and monitoring microbiome health is that it measures holistic dynamics of the ecological system without focusing on specific taxa. Hence, thresholds for within-host gut microbial ß-diversity stability may provide a more consistent measure of ecosystem health across a wider variety of human populations. However, host phenotypic context is required when interpreting stability, as some gut microbiome drifts in response to changing dietary habits and host physiology may be favorable, indicative of an adaptation of the gut microbiome to its changing environment. For example, a recent study from our research group has demonstrated that gut microbiomes become increasingly divergent, or unique, to each individual with age. This adaptation of the gut ecosystem with aging may be favorable, as it was associated with increased survival in the latest decades of human life.^84^

## Defining microbiome health through its functional capacity

**Figure 1.**
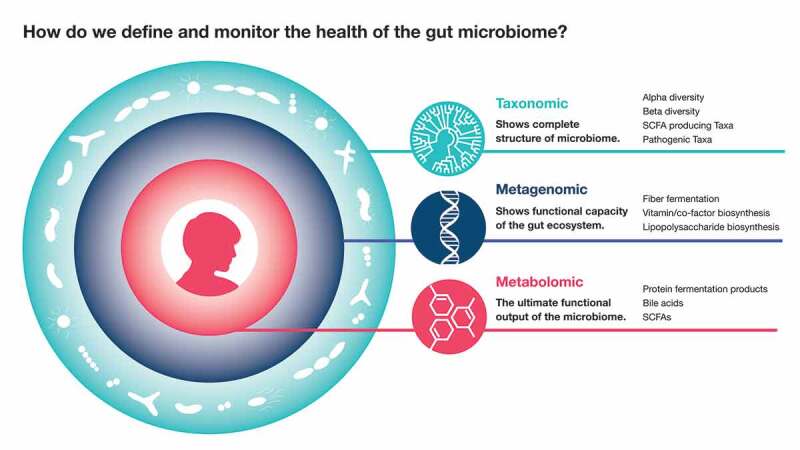
Approaches to defining and monitoring gut microbiome health

While there is considerable inter-individual heterogeneity in gut microbiome composition, the collective functional capacity of the gut microbiome, inferred from its metagenomic content, tends to show greater similarity across individuals^85^ (Figure 1). By some estimates, individuals share twice the number of metabolic pathways (82%) than they do species (43%) in the gut, as measured by metagenomic shotgun sequencing.^86^ Understanding this common functional capacity, and defining a minimal set of essential metabolic and signaling functions, could prove to be a more robust approach to defining microbiome health.

While taxonomic/metagenomic markers help define and monitor healthy gut microbiome dynamics and functional capacity, metabolomics measurements are the ultimate functional output of the ecosystem that influences host physiology. Blood/fecal metabolites are hence the closest measurement to the actual host phenotype, and may potentially constitute a highly translatable metric of microbiome health.

Early work characterizing the functional potential of the gut microbiome identified shared metabolic capacity uniformly present across gut metagenomes of both lean and obese individuals.^85^ These shared functions were dominated by amino acid and carbohydrate metabolism, while cell signaling and membrane transport functions were more variable and distinct across samples. Studies in larger cohorts have since characterized additional shared gut metagenome functions, including cofactor and vitamin biosynthesis, ATP synthesis, amino acid synthesis, and degradation of complex sugars and glycans.^28,87^ The identified functional pathways occurred in similar abundances within each ecosystem, despite considerable taxonomic variability across the same samples. While a number of the identified metabolic pathways have been attributed to common housekeeping functions (e.g. ATP synthesis), others (vitamin biosynthesis and glycan degradation) point to specific roles fulfilled by the gut microbiota that are essential for ecosystem health.^22^

An alternative to defining a core functional capacity across a healthy population is to focus on microbiome functions most perturbed in diseased individuals. Evidence to date suggests that functional signatures of disease in the gut microbiome are often shared across disease states. For example, stool metagenomic sequencing studies revealed that certain functional modules (groups of genes involved in the same biological process), such as lipopolysaccharide biosynthesis, modules involved in iron transport, and enzymes involved in redox balance have been shown to be consistently enriched across multiple diseases.^88,89^ This could be due in part to the shared underlying phenotypic features across diverse disease states, like inflammation. Similarly, a decline in the capacity to synthesize SCFAs by the gut microbiome has been linked to inflammation, type II diabetes, and related metabolic disorders.^90^ Focusing on these common functional shifts within the gut microbiome across diseases may be particularly useful in defining more universal markers of gut microbiome health. For example, an optimal range for SCFA production capacity could more broadly translate across populations with taxonomically variable microbiomes, as different taxa may be responsible for SCFA production in different individuals. Furthermore, by identifying nonspecific functional disease signatures in the gut microbiome, we will be able to isolate disease-specific functional perturbations as well.

Despite its promise, there are several challenges to relying on functional capacity for defining gut health. Even with a greater proportion of gut microbiome function being shared between individuals, there is still considerable variability in gut metagenomic capacity between individuals and across cohorts.^88,91^ These differences are likely a result of selective pressures from variable dietary and environmental factors across populations,^92^ and highlight a degree of personalization of the microbiome to its individual host. Furthermore, metagenomic capacity is not equivalent to ecosystem function, as the presence of a gene does not necessarily mean it is actively being transcribed and translated into a functional protein. Some of these limitations will be overcome by gut microbiome transcriptomic and proteomic profiling approaches, with growing data from large and diverse cohorts utilizing these types of platforms. However, even when proteins are quantified directly, the stoichiometric and thermodynamic context of the ecosystem defines the permissibility of certain chemical reactions. Thus, the integration of host physiological, dietary, and lifestyle data will be necessary to gain more detailed insight into *in situ* gut microbial functional dynamics.

## Defining microbiome health through its metabolic products

An alternative to focusing on the gut microbiome’s genetic capacity when monitoring microbiome health is focusing on the gut microbiome’s ultimate functional output: the diverse pool of metabolites and signaling molecules synthesized by the gut microbiota that directly influence host physiological processes^26^ (Figure 1). Bioactive, microbially-derived compounds enter host circulation, are often metabolized by the liver into human-microbial co-metabolites, and exert direct effects on a variety of host organs.^93^ To date, these effects have been shown to be both beneficial and detrimental to human health.

The diverse pool of metabolic compounds produced by the gut microbiota includes SCFAs, secondary bile acids, B-vitamins, a wide variety of protein and phytochemical fermentation products, and several different lipid species (cholesterol derivatives and sphingolipids).^94–96^ SCFAs, such as propionate and butyrate produced through the fermentation of dietary fibers by gut bacteria, are arguably the best studied gut microbial metabolic products. Their synthesis by the gut microbiota is essential for regulating host immune responses and metabolic homeostasis.^97,98^ SCFAs are the main source of energy for colonic epithelial cells^99^ and have been shown to regulate gut-derived incretin hormones, thereby controlling host energy metabolism.^100^ SCFAs further help regulate immune homeostasis through promoting differentiation of regulatory T-cells^101^ and stimulating anti-inflammatory cytokine production in T-effector cells.^102^ A number of different SCFA-recognizing G-protein coupled receptors (GPR) have now been identified, including GPR41 and GPR43, which are expressed across numerous tissues including adipose tissue and the peripheral nervous system.^103,104^ The beneficial effects of SCFAs on host physiology extend beyond immune and metabolic regulation, with recent evidence implicating SCFAs in inhibiting postmenopausal and inflammation-induced bone loss^105^ and promoting muscle mass and function.^106,107^ SCFAs have further been implicated as protective against certain neurodegenerative disorders, including Alzheimer’s and Parkinson’s disease,^108,109^ although contradictory results have also been reported in some animal studies.^110,111^ Thus, gut microbial SCFA production regulates a diverse number of host processes.

Though perhaps studied less extensively than SCFAs, microbial protein fermentation products also play key roles in regulating host physiology. Tryptophan catabolites including indole, indole-3-propionate and indole-3-acetate have been shown to bind the Aryl hydrocarbon receptor (AHR)^112^ and regulate host immune responses, reducing liver and intestinal inflammation as well as promoting gut barrier function in mouse models.^113–115^ Indole dependent AHR activation was shown to increase host-derived IL-22 production, thereby lowering inflammation and contributing to healthy mucosal function.^116,117^ Consistently, depletion in gut microbial capacity for indole synthesis has been recently reported in a number of inflammatory conditions including celiac disease,^118^ aging,^119^ and obesity.^120^

Microbial breakdown products of phosphatidylcholine and carnitine, particularly trimethylamine converted in the liver to TMAO, are commonly elevated in individuals on diets high in red meat.^121^ TMAO has been shown to promote cardiovascular disease in animal models,^122^ and is associated with increased risk of cardiovascular disease in humans.^123,124^ The histidine microbial catabolite imidazole propionate was recently shown to be more readily synthesized by gut microbiomes of type II diabetic patients relative to controls.^125^ Imidazole propionate further promoted insulin resistance in mice via downregulating insulin receptor substrates 1 & 2 in liver, muscle, and adipose tissue, highlighting its role in regulating metabolic homeostasis through its action on distal organs. Other protein fermentation byproducts, including *p*-cresol sulfate and phenylacetylglutamine, have been identified as potential toxins at high enough plasma concentrations, promoting cardiovascular disease through several mechanisms.^69,126^ The gut microbiome is further capable of synthesizing all eight essential B-vitamins, and is a major contributor to the total B-vitamin pool available to its host.^127^ B-vitamins play an integral role as co-factors in metabolic reactions and regulators of host immune cells, being yet another point of cross-talk between the gut ecosystem and host physiology.^128^ Thus, measuring microbiome derived small molecular weight compounds appears to be key to our understanding of healthy gut microbiome-host dynamics.

A common characteristic of gut microbial metabolism is its funnel-like effect, where a diverse group of dietary substrates gives rise to a relatively small number of microbial breakdown products.^129^ These substrates include amino acids (tryptophan, phenylalanine, tyrosine), polysaccharides, and dietary phytochemicals. In the case of phytochemicals, at least 4000 unique compounds have been identified in fruits, vegetables, wholegrains, legumes and other plant foods.^130^ This extreme chemical diversity of human diets likely contributes to both geographic and inter-individual variability in gut microbiome composition, as many phytochemicals have microbiome-modulatory properties.^131–133^ A number of common breakdown products of these diverse dietary compounds are absorbed by the host and can be measured in the blood. For example, cinnamoylglycine and hippurate (glycine conjugates of microbially produced cinnamate and benzoate, respectively) are readily detected in the blood of conventional, but not germ-free, mice.^134^ These microbial compounds can be synthesized from either aromatic amino acids or a diverse pool of plant polyphenols (i.e. a specific class of dietary phytochemicals, ~700 of which have been identified in foods^135,136^). Hence, multiple diet-microbiome combinations may result in similar blood concentrations of these small molecular weight compounds.

The funneling of diverse diets into a narrower metabolic readout by the gut microbiome can be leveraged to define more universal signatures of gut microbiome health. As an initial proof of this concept, our research group has recently demonstrated that a subset of 40 plasma metabolites can explain up to 50% of variance in gut α-diversity across a cohort of hundreds of individuals, while a smaller subset of 11 primarily microbial metabolites can successfully identify individuals with very low α-diversity measures.^66^ Many of the same blood microbial metabolites have since been validated in a geographically distinct UK based cohort (TwinsUK) and a Dutch cohort of individuals diagnosed with metabolic syndrome.^137,138^ Overall, these results provide evidence that blood metabolic signatures of gut microbiome health may be highly stable and consistent across populations with diverse microbiome compositions, diets, and lifestyles.

Fecal metabolites may provide additional insight into gut microbiome health, with recent evidence indicating a high level of correspondence between gut microbiome function and fecal metabolite profiles.^86,139^ Some studies have also shown diagnostic value in measuring fecal metabolites for several diseases including IBD^89^ and early colorectal cancer detection,^140^ which correlated with changes in gut microbial composition across the same disease states. While there is considerable overlap between blood and fecal metabolites,^139^ many of the compounds measured in feces are not absorbed by the host and do not enter circulation. For example, the poorly absorbed fecal microbial metabolite coprostanol was recently shown to reflect the gut microbiota’s capacity for cholesterol metabolism. Higher fecal coprostanol concentrations reflected greater microbial metabolism of cholesterol by microbial cholesterol dehydrogenases, thereby lowering cholesterol availability to the host and improving blood lipid profiles.^96^ Hence, combined metabolomics approaches analyzing both feces and blood may yield more specific and detailed insight into exactly how the gut microbiome contributes to modulating host phenotypes.

## Defining microbiome health through mechanistic modeling

While metabolomics provides a window into the functional output of the gut microbiome, it is limited in its measurement of important metabolites that are rapidly transformed or excreted from the system. Locally produced and consumed metabolites that impact host physiology cannot be captured by measuring standing concentrations of blood and fecal metabolites alone (e.g. SCFAs are rapidly consumed/transformed by both host and microbial cells within the gut). To this end, other analytical methods may be particularly useful. Genome-scale metabolic modeling is an established method successfully implemented to address biological, microbiological, and bioengineering questions.^141,142^ It relies on detailed stoichiometric reconstructions of metabolic networks and calculates the flux of metabolites through those networks given a set of environmental constraints, providing insight into metabolic production, consumption and overall growth.^143^

Over the past several years metabolic modeling has increasingly been adapted to gut microbiome research.^144,145^ The recent release of AGORA has provided the research community with metabolic reconstructions of close to 800 individual gut bacterial strains.^146^ Further methods have since been developed that more accurately infer growth rates of individual taxa within the gut ecosystem, allowing for more precise constraints on ecological simulations.^147^ The advantage of using genome-scale metabolic modeling to define microbiome function is that one can look at the gut ecosystem holistically to infer its overall metabolic capacity under a wide variety of environmental constraints (e.g. redox states, pHs, etc.). This is a major advantage over simply correlating host health with genes or metabolites, as the environmental context of the ecosystem can elicit variable metabolic outputs from the same gut microbiome composition. Models further allow for incorporation of individual diets and medications, allowing for *in silico* adjustments for diet/lifestyle variation within and across individuals, to simulate ecosystem function under different environmental regimes.

The combination of individual genome-scale metabolic models with bacterial abundances in varying host environments has been used to map metabolite abundances onto bacterial metabolism^148,149^ . Recent studies have also attempted to combine genome-scale metabolic models to simulate complex, real-world microbial communities, yielding metagenome-scale metabolic models. This approach is often more challenging, as it requires a careful weighting of community-wide biomass production with individual bacterial population growth. Despite the challenges involved, metagenome-scale metabolic models of microbial consortia have been used to study the interactions between diet, the gut microbiome and SCFA production.^144^ A recent implementation of metagenome-scale metabolic modeling called MICOM demonstrated lower SCFA production fluxes in diabetic individuals compared to healthy controls.^147^ MICOM was also used to demonstrate the potential for metabolic models in predicting personalized prebiotic and probiotic intervention strategies to improve SCFA production. In another study, manually curated AGORA models were used to identify taxa contributing to bile acid deconjugation and described the metabolic bottlenecks during primary bile acid metabolism.^150^ There are also ongoing efforts to integrate gut microbiome metabolic models with host tissue-resolved metabolic models, allowing for a more complete representation of metabolic fluxes across the host-microbiome interface.^151^

Metagenome-scale metabolic models are of particular interest to many researchers as they allow for true “n-of-1” studies, where dietary inputs or the microbial composition of a single sample can be altered *in silico* and the effect on the production of metabolites of interest can be tracked and potentially optimized. Together with the incorporation of personalized dietary estimates, this approach can be used to rationally design personalized interventions that leverage the gut microbiota to achieve a specific functional output aimed at improving host health (e.g. higher butyrate production). However, these approaches still require extensive empirical validation in order to understand which metabolic objectives are predicted accurately by metabolic modeling and which are not.

## Future directions: from diagnosis to personalization

While the various health markers discussed in this review provide a set of measurable targets for potentially optimizing gut microbiome function, finding paths toward rationally engineering the gut microbiome to modify these markers is a far more challenging task. Each person’s gut microbial composition, genotype, physiology, environmental exposures, and dietary habits can dictate the efficacy of any given microbiome-based intervention. Thus, building sophisticated mechanistic models that help integrate this complexity and provide actionable possibilities will play a critical role in harnessing the power of the gut microbiome to influence human health.

To date, attempts to modulate gut microbiome composition in humans to treat disease have had promising, but limited success. While fecal microbiota transplantation (FMT) has been extremely effective in treating rCDI,^152^ the same approach has been less successful in treating other gastrointestinal disorders, such as IBD.^153^ This is likely due to the fact that rCDI is driven by the dominance of a single invasive pathogen that is normally suppressed by a healthy commensal microbiota. The etiologies of many other microbiome-related diseases are likely more complex, involving networks of bacterial species and metabolites, which will require much more targeted, and possibly personalized, interventions.

Prebiotic, probiotic, and synbiotic (i.e. a combination of pre- and probiotics) forms of interventions have been employed to engineer the functional output of the human gut microbiome and improve host health. For example, a combined supplementation of *Lactobacillus acidophilus, Lactobacillus casei*, and *Bifidobacterium bifidum* with inulin for 12 weeks in type II diabetic patients undergoing dialysis resulted in improved metabolic (fasting plasma glucose and insulin) and inflammatory (C-reactive protein) markers.^154^ Similar synbiotic interventions were shown to significantly increase self-reported measures of gastrointestinal wellbeing and improve scores on IBD Questionnaires among UC patients.^155,156^ Although promising,^157^ the efficacy of any one of these interventions is often highly dependent on both the physiology and the baseline gut microbial composition of each individual. For example, Chung *et al*.^158^ recently demonstrated that supplementation with wheat bran arabinoxylan oligosaccharides in healthy adults induced variable microbiome changes dependent on the baseline relative abundance of *Prevotella*. In a different study, Baxter *et al*.^159^ demonstrated that feeding healthy young adults with degradable fibers (resistant potato starch (RPS) and inulin) had variable impact on gut microbiome composition and butyrate production. RPS supplementation resulted in increased relative abundance of *Ruminococcus bromii* and *Clostridium chartatabidum*, but only in a subset of participants. Interestingly, those participants who demonstrated an increase in these two taxa showed the greatest benefit from the intervention, as measured by increased fecal butyrate levels.

**Figure 2.**
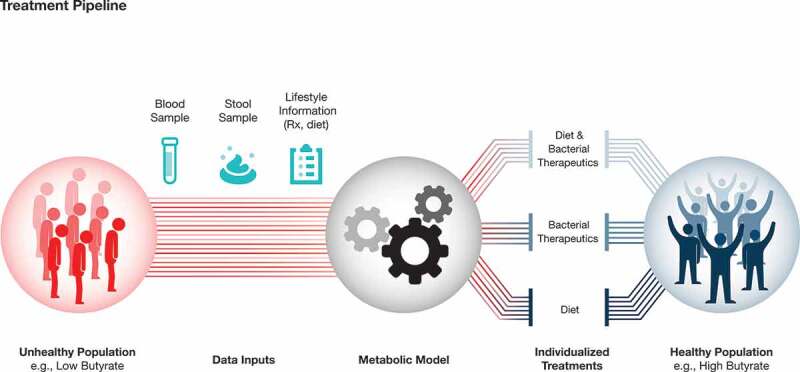
Personalized interventions for optimizing gut microbiome health

Not only is there heterogeneity in the ability to modify the gut microbiome based on its initial composition, evidence suggests that gut microbiome composition may also dictate the efficacy of treatments targeting other aspects of human health. Hjorth *et al*.^160^ demonstrated that a person’s gut microbial *Prevotella*-to-*Bacteroides* (P/B) ratio predicted their response to a high fiber diet weight loss program. While individuals with a high P/B ratio lost more weight on a higher fiber diet relative to an average Danish diet, individuals with a low P/B ratio did not see any added benefit from the same high fiber intervention. Similarly, Zeevi *et al*.^15^ demonstrated that individuals have highly variable postprandial glycemic responses when consuming the same foods. Incorporation of gut microbiome data into their algorithm allowed them to improve predictions of personalized glycemic responses to different dietary components. Collectively, these studies indicate that a better understanding of gut microbial function may lead to highly personalized interventions aimed at altering the composition of the microbiome, but also optimizing treatment strategies that leverage the existing metabolic capacity of an individual’s microbiota (Figure 2).

Metagenome-scale metabolic modeling allows for personalization of gut microbiome interventions. Blood and stool samples, coupled with lifestyle/dietary information, are used to build and constrain personalized microbiome models for each individual. Thousands of *in situ* simulations of possible microbiome modulating interventions are subsequently performed. The optimal personalized intervention is chosen, resulting in more effective treatment.

**Figure 3.**
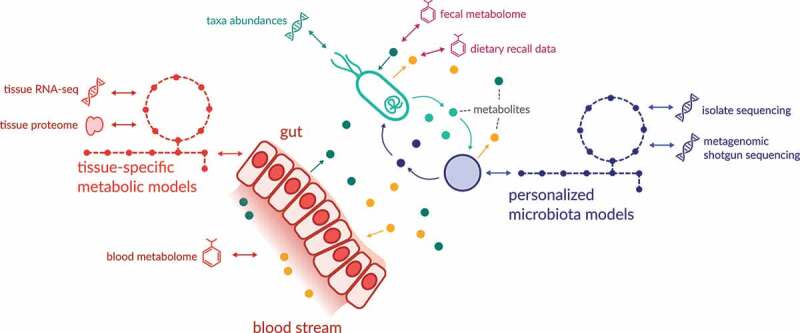
Integration of different data types into mechanistic models

Metagenome-scale metabolic modeling provides a unique approach toward integration of host dietary, physiological and gut microbial data in order to design personalized ecological therapeutics that predictably modulate gut microbiome function. These models incorporate existing metabolic reconstructions of gut microbial strains,^146^ personalized chemical stoichiometric inputs based on self-reported diet, growth rate tradeoffs among microbes in the ecosystem, and can be further constrained through compartmentalization and adding host physiological processes (for example a liver and kidney compartment). This enables cost-efficient testing of thousands of potential interventions for a single individual and provides a path toward the rational design of pro- and prebiotics with a high likelihood of success. For example, the objective of an intervention may be to promote higher gut butyrate production in individuals suffering from IBD. First, fecal sequencing data for each individual is paired with their blood and fecal metabolomics data. A personalized metabolic model is generated for each individual, which is further constrained by dietary inputs inferred from self-reported dietary questionnaires. Such an approach can also contextualize microbiome health insights using metabolic reconstructions for host tissues which are built from host proteome or transcriptome data. These tissue-specific models can then be used to describe metabolic host-microbiome interactions (e.g. utilization of SCFAs by colonic cells). Dietary data is key to defining a personalized growth environment for each individual microbial community, while blood and fecal metabolites help both optimize the model and validate the accuracy of its predictions (Figure 3). Once a personalized metabolic model with integrated metabolomics and dietary data is generated for each individual, thousands of simulations are subsequently performed to identify dietary, prebiotic or probiotic interventions that yield highest butyrate production. These predictions are done by simulating dietary spike-in experiments into the individual models. Similarly, a bacterial strain (probiotic) may be introduced into the model, and its effect on butyrate production tested. While one person’s gut microbiome may only require inulin or pectin supplementation to produce butyrate, another may require a specific bacterial strain to be introduced into the ecosystem to reestablish the metabolic capacity for butyrate production within the gut microbiota (Figure 2).

Sequencing data is used to determine the presence or absence of microbial genes and enzymes in a given model. Transcriptional and proteomics data from host tissues are used in constructing tissue-specific metabolic models. Fecal and blood metabolomics are used to constrain model parameters, validate model predictions, and to assess available growth niches in the gut microbiome. The output of the model is a prediction of all metabolic fluxes (mass conversion per unit of time). Dots denote exchanged metabolites. Metabolic models are represented by stylized pathway diagrams.

However, predictions generated by metagenome-scale metabolic models are only as good as the model itself. Thus, large-scale quantitative validation experiments are necessary for testing these models, where *in* *vivo* microbial growth rates and metabolite fluxes are measured to identify which metabolic processes are captured well by the model and which ones are not. *Ex vivo* growth experiments, where fecal samples are incubated under anaerobic conditions with a wide array of dietary metabolites, represent a promising high-throughput approach to observing gut microbial community metabolic output in the absence of the host^161,162^ . Similarly, humanized gnotobiotic mice may prove to be a key tool in improving metagenome-scale metabolic models, by providing a well-controlled *in* *vivo* experimental model to optimize metabolic predictions.^163^ Ultimately, these predictions will need to be tested in human intervention trials, as the final stage of clinical translation.

### Conclusion

Rapid progress in human gut microbiome research over the last decade has greatly expanded our understanding of what constitutes a healthy gut ecosystem. With a growing focus on the functional potential of the gut microbiome, and the measurement of microbial metabolic products, establishing quantifiable and translatable metrics for monitoring the gut ecosystem seems within reach. However, deepening our collective understanding of gut microbiome health is only the first step toward treating microbiome-related illnesses. Effective engineering of the gut ecosystem toward a desirable healthy state is the ultimate goal for researchers and clinicians alike, and continues to be a major challenge in the field. Integrative methods that can accurately model complex gut microbiome-diet-host interactions may help expand our understanding of the human gut microbiome, and open new possibilities for designing personalized therapeutic interventions.
